# Novel Design of Co-Poly(Hydrazide Imide) and Its Complex with Cu(I) for Membrane Separation of Methanol/Dimethyl Carbonate Mixture

**DOI:** 10.3390/membranes13020160

**Published:** 2023-01-27

**Authors:** Galina Polotskaya, Nadezhda Tian, Ilya Faykov, Mikhail Goikhman, Irina Podeshvo, Nairi Loretsyan, Iosif Gofman, Konstantin Zolotovsky, Alexandra Pulyalina

**Affiliations:** 1 Institute of Macromolecular Compounds, Russian Academy of Sciences, Saint Petersburg 199004, Russia; 2 Institute of Chemistry, Saint Petersburg State University, Saint Petersburg 198504, Russia

**Keywords:** membrane, pervaporation, methanol/(dimethyl carbonate) mixture, poly(2,2′-biquinoline-6,6′-dicarbohydrazide)-co-(bistrimelliteimide)methylene-bisanthranylide, metal–polymer complex

## Abstract

Poly(2,2′-biquinoline-6,6′-dicarbohydrazide)-co-(bistrimelliteimide)methylene-bisanthranylide (PHI) and its metal–polymer complex PHI-Cu(I) containing several types of functional groups (hydrazide, carboxyl, amide, and imide fragments) were synthesized to prepare two types of dense nonporous membranes. The study on morphology using scanning electron microscopy (SEM), measurements of mechanical, thermal, and transport properties of the membrane samples was carried out. The main mechanical properties of both membranes do not differ significantly, but the values of ultimate deformation differ palpably as a result of a non-uniform character of the deformation process for the PHI membrane. The thermal analysis based on the curves of thermogravimetric (TGA) and differential thermal (DTA) analyses of the PHI and PHI-Cu(I) membranes revealed peculiarities of the membrane structure. Transport properties were studied in pervaporation (PV) of methanol (MeOH) and dimethyl carbonate (DMC) mixtures including an azeotropic point. Intrinsic properties of the penetrant–membrane system were also determined. It was found that the total flux is higher through the PHI membrane, but the PHI-Cu(I) membrane exhibits a higher separation factor. Calculation of the pervaporation separation index (*PSI*) allowed to conclude that the PHI-Cu(I) membrane exhibits better transport properties as compared with the PHI membrane.

## 1. Introduction

Modern requirements for environmental protection include the use of clean and safe solvents in technological processes. Dimethyl carbonate (DMC) has attracted much attention as a “green” reagent and solvent with low corrosion characteristics [1]. DMC is an important raw material in organic synthesis for carbonylation and methylation of functional monomers in industrial coatings and pharmaceutical industries [2,3]. DMC has been widely used on a large scale as a precursor for polycarbonate resins [4]. High-purity DMC can enhance stability and extend the service life of lithium ion cells [5,6]. DMC is also an excellent antiknock agent of gasoline or diesel fuel. The obvious advantage of DMC over other potential fuel additives (methyl tert-butyl ether or ethyl tert-butyl ether) consists in its slow decomposition into carbon dioxide and methanol (MeOH), which do not cause serious consequences when DMC is released into the environment [7,8,9]. Thus, DMC is fairly positioned as a “new base stone of chemical engineering” in the 21st century [10].

At present, DMC is manufactured using carbon dioxide-MeOH synthesis, MeOH oxidation-carbonylation, and urea alcoholysis [11,12]. In most cases, MeOH is used as a feedstock, and an excess of MeOH is taken to shift the equilibrium of a reaction and increase the yield [13]. During the reactions, MeOH forms an azeotropic mixture with DMC, which cannot be separated by means of conventional distillation [14]. This problem can be solved using membrane technology–pervaporation. Currently, membrane methods are among the most promising for various modern applications [15,16,17,18,19,20,21]. PV is widely used to separate azeotropic mixtures as well as liquids with close boiling points [22,23,24,25,26]. The undoubted advantages of PV are high separation efficiency, low pollution, safe operation, and low power consumption [27]. Successful realization of the pervaporation separation is possible only under the correct choice of a membrane material [28,29]. Up to date, various membrane materials have been studied for the separation of MeOH/DMC mixtures by pervaporation [30,31,32,33,34,35,36]. Hydrophilic polymers such as polyacrylic acid, polyvinyl alcohol [30,31], or chitosan [32,33,34,35] have been shown to be preferentially permeable toward MeOH in pervaporation of MeOH/DMC mixtures. A membrane with a silicone rubber/nanosilica hybrid active layer has been recently developed [10], which showed the preferential permeation of DMC during the pervaporation separation with inverse DMC/methanol selectivity.

Membranes based on polymers of a heteroaromatic structure have attracted a particular interest due to their high level of chemical and structural stability, heat resistance, and mechanical strength [36]. Polyheteroarylene membranes for the separation of liquid mixtures by pervaporation have been reported for various applications [37,38,39,40] except for the separation of a MeOH/DMC mixture.

A representative of the poly(biquinoline dicarbohydrazide)-co-(bistrimelliteimide) has been considered promising for studying it as a membrane polymer [41]. In our previous work, the similar polymer {poly(2,2′-biquinoline-4,4/-dicarbohydrazide)-co-(bistrimelliteimide) methylene-bisanthranylide} was synthesized, which proved to be effective in the separation of the benzene/isopropanol mixture by pervaporation [42].

It is known that membrane characteristics can be effectively controlled by changing the rigidity of a polymer chain, i.e., introducing additional rigid or hinged fragments into a polymer [43]. Another approach to changing the flexibility of a polymer chain is to introduce aromatic fragments into the polymer chain, in which the arrangement of substituents increases or decreases the number of possible conformations and changes the flexibility of the chain. We have previously used this approach in the synthesis of polymers containing 2,2’-biquinoline fragments, which provided free rotation around a single bond connecting two quinoline rings [42]. The polymer chains containing 2,2’-biquinoline fragments, in which functional substituents are located in the positions 4,4′, acquired the ability to form new conformations, i.e., to have kinetic flexibility. Moreover, the intermolecular distance between the polymer chains can be regulated through the formation of metal–polymer complexes, in which a transition metal atom plays the role of a crosslinking site [44,45].

In the present work, a new biquinoline monomer was synthesized. The substituents in this monomer are located in the positions 6,6′, due to which the structure of macromolecules becomes close to a rigid rod and the rotation around a single bond does not lead to an increase in the set of conformations. The goals of the work included the synthesis of PHI and its metal–polymer complex PHI-Cu(I) containing several types of functional groups (hydrazide, carboxyl, amide, and imide fragments) and further preparation of dense nonoporous membranes. Transport properties of these membranes were studied in the separation of a MeOH/DMC mixture by pervaporation. The structure, physical, and mechanical properties of the samples were determined in order to obtain comprehensive information about the novel polymer films.

## 2. Materials and Methods

### 2.1. Materials

Methylene-bisanthranilic acid, N-methylpyrrolidone (NMP), and cuprous chloride were purchased from Sigma-Aldrich (Schnelldorf, Germany) and used without additional purification

### 2.2. Synthesis of Polymers

**Figure 1:**
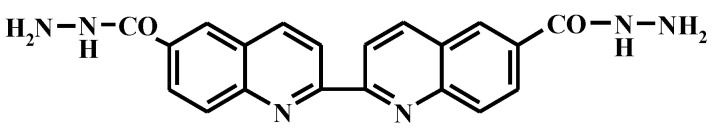
Structure of 2,2/-biquinoline-6,6/-dicarbohydrazide.

Novel bifunctional monomer 2,2′-biquinoline-6,6′-dicarbohydrazide (Figure 1) was synthesized according to the procedure described in [42]. The structure of the synthesized monomer was confirmed using 1H NMR spectroscopy.1H NMR (DMSO-d6) d (ppm) 4.45 (s, 4H), 8.88 (d, 2H), 8.79 (d, 2H), 8.70 (d, 2H), 8.29 (d, 2H), 8.25 (s, 2H), 10.10 (s, 2H).

**Figure 2:**
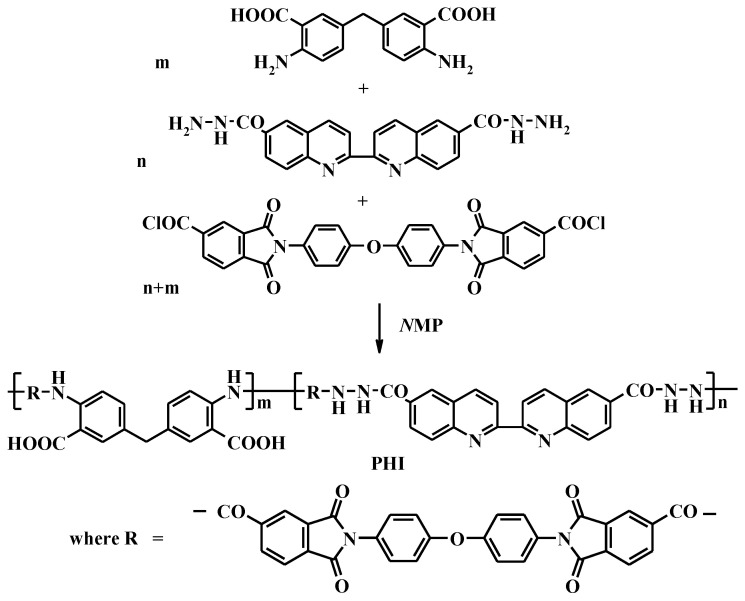
Synthesis of PHI.

N,N’-diphenyloxide-bistrimelliteimide acid dichloride was synthesized according to the procedure described in [42]. Synthesis of poly(2,2′-biquinoline-6,6′-dicarbohydrazide)-co-(bistrimelliteimide) methylene-bisanthranylide (PHI) was carried out using methylene-bisanthranilic acid and 2,2′-biquinoline-6,6′-dicarbohydrazide in a ratio of 80:20 (mol.%) as well as N,N’-diphenyloxide-bistrimelliteimide acid dichloride by low-temperature polycondensation (Figure 2).

**Figure 3:**
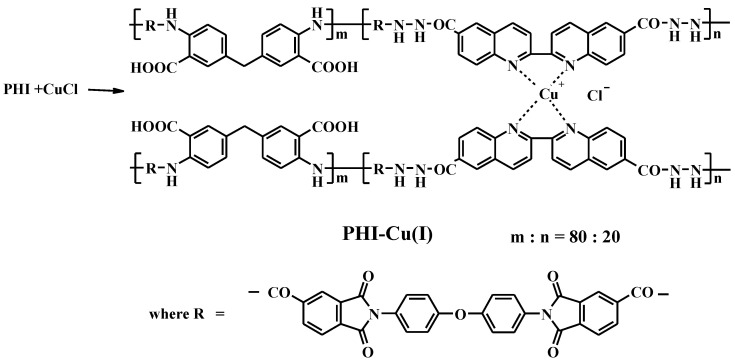
Synthesis of PHI-Cu(I).

Synthesis of metal–polymer complex PHI-Cu(I) was carried out from the PHI copolymer by the interaction with cuprous chloride; NMP was used as a solvent (Figure 3).

### 2.3. Membrane Preparation

Dense flat nonporous membranes based on PHI and PHI-Cu(I) were obtained by casting a 10 wt% polymer solution in NMP on a glass plate. The solvent was evaporated at 140 °C in air. The resulting films were dried in vacuum at 90 °C for about 2 weeks until a constant weight was reached. The thickness of the obtained membranes was measured by a digital micrometer produced by TECHRIM (St. Petersburg, Russia) and was 30–40 μm, and the error of measurements was ± 0.1 μm.

### 2.4. Membrane Characterization

Membrane morphology was studied by scanning electron microscope SEM Zeiss SUPRA 55VP (Carl Zeiss AG, Oberkochen, Germany). Before tests, a graphite layer of 20 nm in thickness was coated on the sample surface by cathode sputtering, using the Quorum 150 (Great Britain) setup.

The film density *ρ* was estimated using the flotation method with a laboratory-made measurement unit. The mixture of toluene and carbon tetrachloride was used to equilibrate the specimens at 20 °C (*ρ*_Toluene_ = 0.867 g/cm^3^, *ρ*_CCl4_ = 1.594 g/cm^3^). The samples of 0.05–0.10 g were used; the error of measurements was ± 0.0001 g/cm^3^.

An AG-100kNX Plus setup (Shimadzu, Kyoto, Japan) operating in a uniaxial extension mode was used to study the mechanical characteristics of the membranes. Strip-like samples, which were 2 mm wide and 30 mm long, were stretched at room temperature at a rate of 10 mm/min, according to ASTM D638 requirements. The Young’s modulus, E, the yield stress, *σ_y_*, the break stress, *σ_b_*, and the ultimate deformation, *ε_b_*, were determined. These characteristics of each material tested were obtained by averaging the test results of seven samples.

The thermogravimetric (TGA) and differential thermal (DTA) analyses were performed to characterize thermal stability of the films tested. A DTG-60 thermal analyzer (Shimadzu, Kyoto, Japan) was used, samples (~5 mg) being heated in air up to 600 °C at a rate of 5 °C /min.

Sorption experiments were performed by immersion of membrane samples in individual liquids (MeOH and DMC) at atmospheric pressure, 25 °C. After a certain time, samples were removed from the liquid, carefully wiped with tissue paper and immediately weighed; the error was ± 10^–4^ g. The experiment was continued until sorption equilibrium was reached. 

The degree of equilibrium sorption *S* (g liquid/100 g polymer) was calculated by the equation:

(1) $$S=\frac{M_{S}-M_{d}}{M_{d}}$$

where *M_S_* is the weight of a swollen membrane upon the equilibrium state and *M_d_* is the weight of a dry membrane.

Desorption experiments were carried out at the atmospheric pressure and temperature of 25 °C. Kinetic curves of desorption were plotted in the coordinates *M_t_/M_∞_ = f(t^1/2^/l)*, where *M_t_* is the amount of a desorbed substance per time *t*, *M_∞_* is the equilibrium amount of a desorbed substance, which was determined as a difference between the weight of a swollen membrane and the weight of a membrane dried to a constant weight, and *l* is the membrane thickness [46]. The effective diffusion coefficient *D* was calculated by the equation:

(2) $$D=\pi /16\times tg^{2}\beta$$

where *tgβ* is tangent of the initial linear slope of the desorption kinetic curves when *M^t^ = M*/<0.4.

### 2.5. Pervaporation Tests

Membrane transport properties were evaluated using a pervaporation setup with an effective membrane area of 14.8 cm^2^ at 40 °C. A downstream pressure below 10^−2^ mm Hg was maintained. The stationary flow in these conditions was established after 5–6 h, and the membranes showed a stable result for a month. A non-flowing type cell made of stainless steel and equipped with a stirrer was used. The permeate was collected into a trap cooled with liquid nitrogen, weighted, and analyzed. The experiments were carried out at least 3 times for each of the feed compositions and the measurement errors did not exceed 1%. The permeate composition was determined using a chromatograph Chromatec–Crystal 5000.2 (Chromatec, Russia) equipped with a thermal conductivity detector and a column Porapak Q 80/100 mesh.

The separation factor was calculated by the equation:

(3) $$\alpha_{MeOH/DMC}=(Y_{MeOH\;}/Y_{DMC})\;/\;(X_{MeOH}/X_{DMC})$$

where *Y_MeOH_* and *Y_DMC_* are the weight fractions of MeOH and DMC in the permeate and *X_MeOH_* and *X_DMC_* are the weight fractions of MeOH and DMC in the feed. 

The total flux through a membrane (*J*) was determined as an amount of a liquid penetrated through a membrane area per a time unit. To compare permeability of membranes with a different thickness *l* varied from 40 to 30 µm, values of the normalized total flux (*J_n_*) was used. *J_n_* is the total flux through a membrane with 30 µm thick calculated as: *J_n_ = J ⋅ l/30*.

The pervaporation separation index (*PSI*), which is a parameter generalizing transport properties of a membrane, was calculated as:

(4) $$PSI=J_{n}\times \left(\alpha_{MeOH/DMC}-1\right)$$

To estimate intrinsic properties of a penetrant–membrane system, permeability and selectivity were calculated [47]. The membrane permeability *P_i_* can be determined as a flux of a component normalized for the membrane thickness and driving force; it was calculated using the following equation:

(5) $$P_{i}=j_{i}\frac{l}{p_{i0}-p_{il}}$$

where *j_i_* is the molar flux of component *i* (cm^3^ (STP)/cm^2^ s), and *p*_*i*0_ and *p_il_* are the partial pressures of component *i* on both sides of a membrane (0 stands for the surface on the feed side, and *l* stands for the surface on the permeate side). Permeability was expressed in Barrer units (1 Barrer = 1×10^−10^ (cm^3^ (STP) cm/cm^2^ s cmHg).

Membrane selectivity (*β_MeOH/DMC_*) was defined as a ratio of permeabilities:

(6) $$\beta_{MeOH/DMC}=P_{MeOH}/P_{DMC},$$

### 2.6. Computational Methods

Preliminary optimization of the molecular geometry was performed with the ChemOffice CS Chem3D Ultra package by methods of molecular mechanics (MMFF94). The full geometry optimization of all model structures was carried out at the GFN2-xTB level of theory [48] with the help of the ORCA 5.0.3 program package [49]. The ground multiplicity state of all model systems is the singlet, and spin-restricted approximation (closed electron shell) was applied. No symmetry restrictions were applied during the geometry optimization procedure. The Hessian matrices were calculated for all optimized model structures to prove the location of correct minima on the potential energy surface (no imaginary frequencies were found in all cases). The electronic structure was refined with the r^2^SCAN-3c method [50]. Post-processing of the obtained wave functions was carried out with Multiwfn 3.8 [51] and Chemcraft.

## 3. Results and Discussion

PHI and its metal–polymer complex PHI-Cu(I) were synthesized using new monomer 2,2′-biquinoline-6,6′-dicarbohydrazide. Novel PHI and PHI-Cu(I) membranes containing several types of functional groups (hydrazide, carboxyl, amide, and imide fragments) were prepared as dense nonporous membranes. The comparative study on their structure, physical, mechanical, thermal, and transport properties was carried out.

### 3.1. Membrane Structure

**Figure 4:**
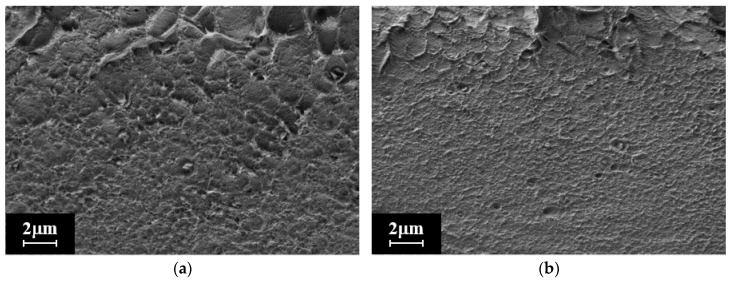
SEM images of film cross-sections: (**a**) PHI and (**b**) PHI-Cu(I).

The morphology of the PHI and PHI-Cu(I) membranes was studied by SEM. Figure 4 shows micrographs of the cross-section of the PHI and PHI-Cu(I) membranes. Both membranes have a dense and defect-free structure, but a difference in the cross-section structures of these films is apparent. The morphology of the PHI-Cu(I) membrane is more dense and uniform through the thickness as compared with the PHI membrane. Some differences in the morphologies of the studied membranes can be explained by the crosslinking of PHI macromolecules through the Cu^+^ ion, which makes the PHI-Cu(I) membranes more dense (as measured by the flotation method, *ρ*(PHI) is equal to 1.368 g/cm^3^ and *ρ*(PHI-Cu(I)) is equal to 1.375 g/cm^3^).

### 3.2. Mechanical Properties

**Figure 5:**
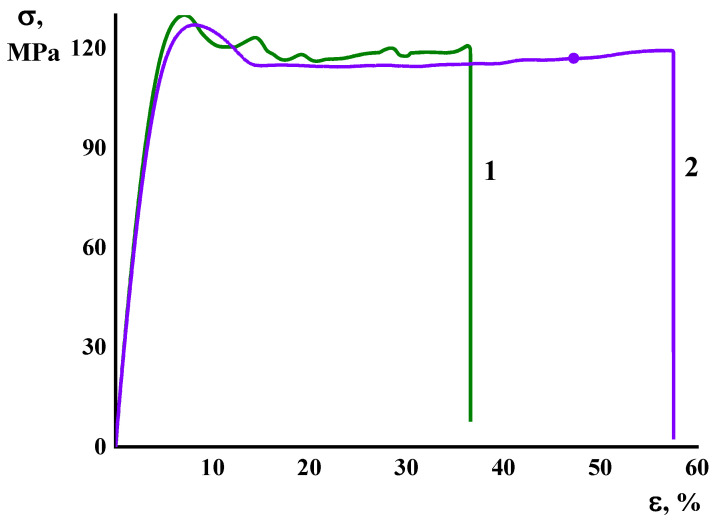
Stress–strain curves of (1) PHI and (2) PHI-Cu(I) membrane films.

Figure 5 demonstrates the stress–strain curves of the PHI and PHI-Cu(I) samples, which imply a plastic character of deformation, since the curves show a local maximum (yield point) and subsequent stress decay corresponding to the formation of the neck. The neck propagation along the samples takes place under the deformation of both the PHI and PHI-Cu(I) membranes until the break point. Table 1 shows data on the Young’s modulus *E*, the yield stress *σ_y_*, the break stress *σ_b_*, and the ultimate deformation *ε_b_* for the PHI and PHI-Cu(I) membranes; the *E*, *σ_y_*, and *σ_b_* values of both membranes do not differ significantly. One can suppose that the formation of the metal–polymer complex should provide the generation of new intermolecular interactions through the Cu^+^ ions (Figure 3). However, the stiffness of the PHI-Cu(I) membrane is yet somewhat less than that of the PHI membrane (Table 1). To treat this discrepancy, we can assume that the formation of complexes between the neighboring chains of PHI produces the local interruptions of the system of intermolecular bonds, which exists in unmodified PHI.

Table 1 shows that only the ultimate deformation (*ε_b_*) values differ palpably for the PHI and PHI-Cu(I) membranes. To treat this difference, we should take into account that both membranes break in the course of the neck propagation across the sample. Thus, the *ε_b_* value reflects an extent of uniformity of the material structure. Figure 4 (Curve 1) shows a non-uniform character of the deformation process for the PHI membrane. During the extension process, several necks are successively formed in different parts of the gauge length of the film. Subsequently, they move along the sample, and over-stress takes place in the cross-section of the sample, where they meet each other provoking the rupture.

### 3.3. Thermal Analysis

**Figure 6:**
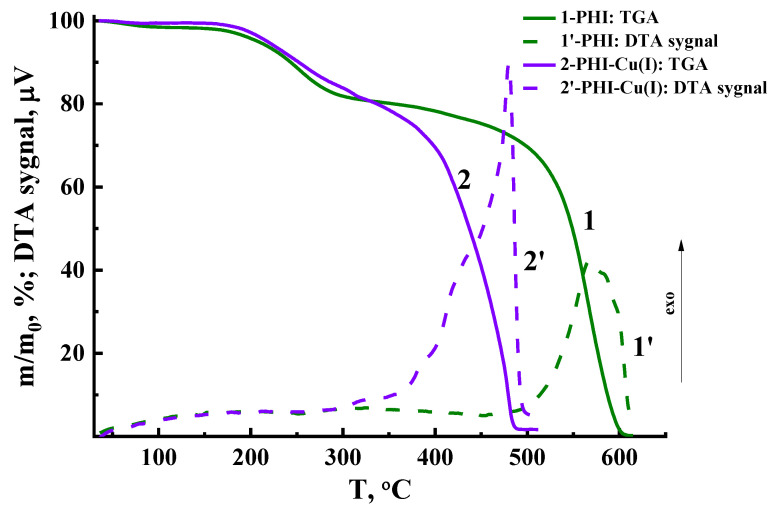
TGA and DTA curves of PHI and PHI-Cu(I) films.

The TGA curves of both the PHI and PHI-Cu(I) membranes reflect the complex character of the processes that take place while heating the films in air atmosphere (Figure 6). A two-stage process of the weight decrease is inherent to both materials. The first one occurs in the temperature range from 140–150 up to 300–320 °C. At this stage of heating, the thermal cyclization of PHI occurs, which leads to the formation of polyoxadiazole and elimination of water. It should be noted that no palpable difference in the behavior of both materials under study was registered at the first stage of heating (Figure 6).

A sharp decrease in the sample weight up to complete volatilization of the polymer occurs at the second stage of heating (above 320–350 °C). At the same stage, an intense exothermal effect was registered by the DTA system (Figure 6). Specifically at this stage, the thermal destruction of the PHI-Cu(I) membrane differs significantly from that of PHI. The process of the weight decrease in PHI-Cu(I) ends up at 490 °C, which is 120 °C below that of the PHI. On the other hand, the exothermal effect in the PHI-Cu(I) sample is shifted to a low-temperature region as compared to PHI (Table 2, Figure 6). The difference in the maximum of the DTA signals for PHI and PHI-Cu(I) is as high as 90 °C.

The TGA data were used to calculate the indices of the thermal stability (*τ_5_* and *τ_10_*), which are the temperature values at which the polymer sample loses 5 and 10 wt% of its initial weight under the thermal destruction processes. Table 2 shows that the thermal stability of the PHI-Cu(I) membrane is lower than that of PHI. This shift of the thermooxidative destruction process in PHI-Cu(I) toward a low-temperature region is obviously provoked by the well-known effect of the catalytic action of copper, oxides, and salts thereof on the destruction of organic compounds. The TGA curve of the PHI-Cu(I) membrane demonstrates the onset of intense destruction at about 300–320 °C. It can be assumed that specifically at this stage of heating, some additional compounds of Cu appear in the polymer volume, thereby catalyzing further intense destruction of the material. Therefore, we can conclude that the above-mentioned temperature characterizes the limit of the thermal stability of PHI-Cu(I).

After completing the thermal destruction process in the PHI-Cu(I) membrane (at above 500 °C), the sample weight remains constant at a level of 0.4% of the initial weight. This “coke residue” characterizes an amount of a copper composition that is formed after the complex dissociation.

### 3.4. Transport Properties

To characterize the transport properties of the PHI and PHI-Cu(I) membranes, the pervaporation separation of a MeOH/DMC mixture was carried out at 40 °C. Some physical properties of the organic liquids under study (MeOH and DMC) are shown in Table 3. It is noteworthy that the molar volume and kinetic diameter of the DMC molecules significantly exceed the corresponding parameters of the MeOH molecules.

The transport of penetrant molecules through a membrane significantly depends on the physical properties of the polymer membrane as such. Table 4 presents the data on the density and sorption capacity of the PHI and PHI-Cu(I) membranes. The membrane density was determined by the flotation method, which revealed that the density of the PHI-Cu(I) membrane is greater than that of the PHI membrane. The formation of the metal–polymer complex should lead to the generation of new intermolecular interactions through the Cu^+^ ions, which, in turn, leads to the structuration of the polymer chains and increases density. 

In the process of pervaporation, the transport properties of the studied membranes are substantially determined by the thermodynamic factor – sorption capacity and solubility of the penetrants in the membrane material. Sorption studies were carried out by immersing membrane samples into individual liquids. It was found that both membranes do not adsorb DMC at all. The polar molecules of MeOH are adsorbed quite extensively. Table 4 shows that the sorption degree and effective diffusion coefficients of MeOH in the PHI membrane is higher as compared to the values of these parameters in the PHI-Cu(I) membrane. 

**Figure 7:**
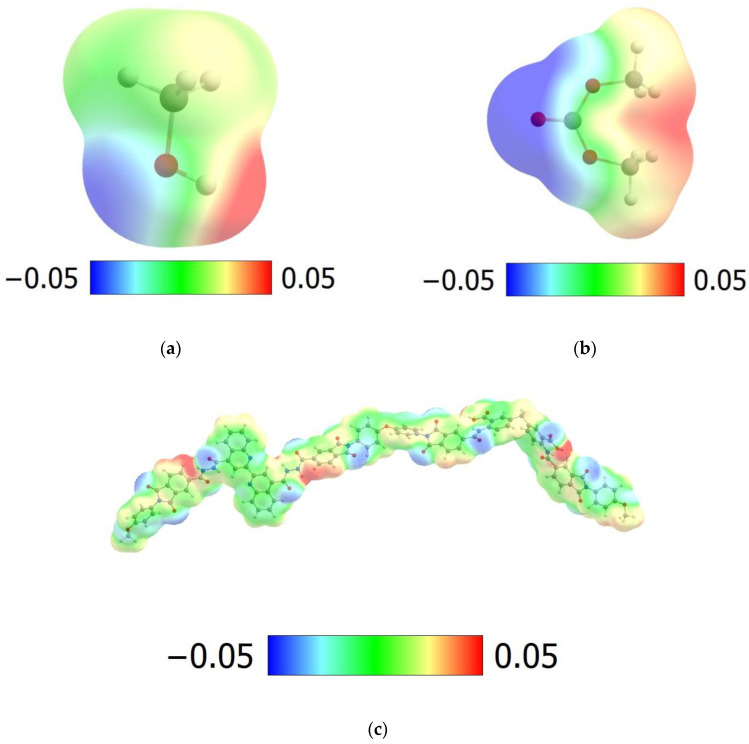

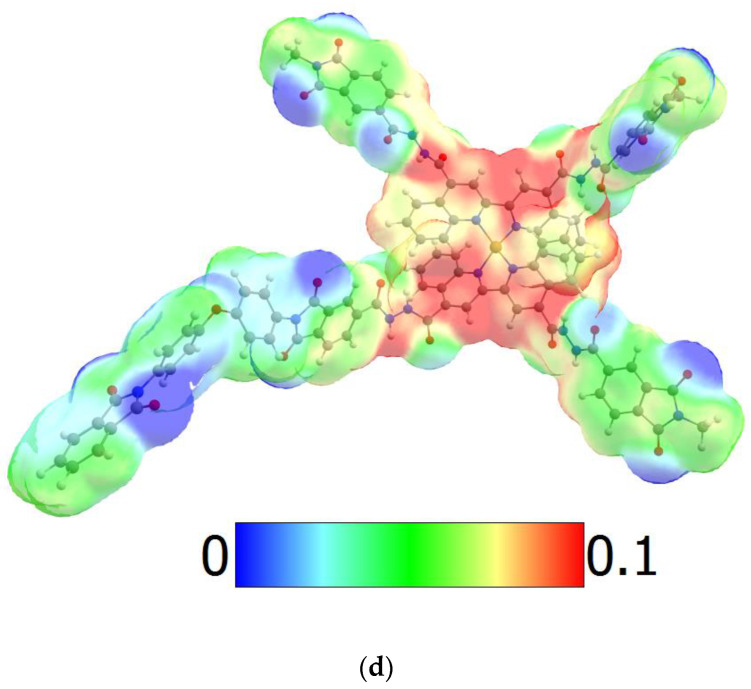
MEP maps for MeOH (**a**), DMC (**b**), dimer (**c**), and Cu-complex with two monomer units (**d**). Contour value: 0.001. Values range: −0.05–0.05 (**a**–**c**), 0–0.1 (**d**).

The results obtained can be explained by the Molecular Electrostatic Potential (MEP) maps, which are widely used for discussing non-covalent interactions. Figure 7 shows the ESP maps for MeOH, DMC, dimer, and Cu-complex with two monomer units. Thus, for MeOH, we observe two localized areas corresponding to the donor (blue area) and acceptor (red area) abilities of O and H atoms, while for DMC, these areas are delocalized. This leads to the worse binding of DMC on the adsorption sites of the polymers.

The lower degree of sorption and a decrease in the diffusion coefficients of MeOH in the PHI membrane compared to the PHI-Cu(I) membrane can be explained by the presence of a large acceptor region near the Cu atom (Figure 7d) in the PHI-Cu(I). This region allows the methanol molecules to remain in place for a significant period of time, changing the diffusion mechanism from jump to random walks over the surface.

**Figure 8:**
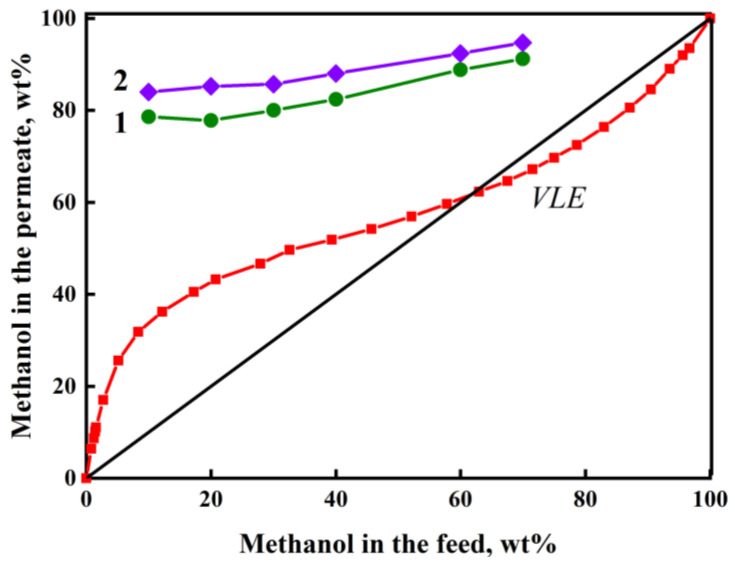
Dependence of methanol concentration in the permeate on methanol concentration in the feed for pervaporation of the MeOH/DMC mixture using the (1) PHI and (2) PHI-Cu(I) membranes, 40 °C. Red line is the curve of vapor-liquid equilibrium (VLE) in the MeOH/DMC system, 40 °C.

Pervaporation was carried out over a wide range of feed concentrations, including the composition of the azeotropic mixture: 61 wt% MeOH and 39 wt% DMC, at 40 °C [52]. Figure 8 shows the dependence of the MeOH concentration in the permeate on the methanol concentration in the feed for pervaporation of the MeOH/DMC mixture using the PHI and PHI-Cu(I) membranes. To compare the results of the pervaporation tests, Figure 8 also shows the vapor-liquid equilibrium (VLE) curve for the MeOH/DMC system, which has an inflection at the azeotropic point [52]. The course of the permeate–feed concentration curves for pervaporation essentially differs from the course of the VLE curve. In contrast to the evaporation process presented by the VLE curve, pervaporation using our membranes produces a permeate that is predominantly enriched with MeOH throughout the entire concentration range of the feed. Moreover, the PHI-Cu(I) membrane produces a permeate with a higher methanol content as compared to the PHI membrane. This difference leads to the fact that the separation factor (*α_MeOH/DMC_*) of the PHI-Cu(I) membrane significantly exceeds that of the PHI membrane. 

**Figure 9:**
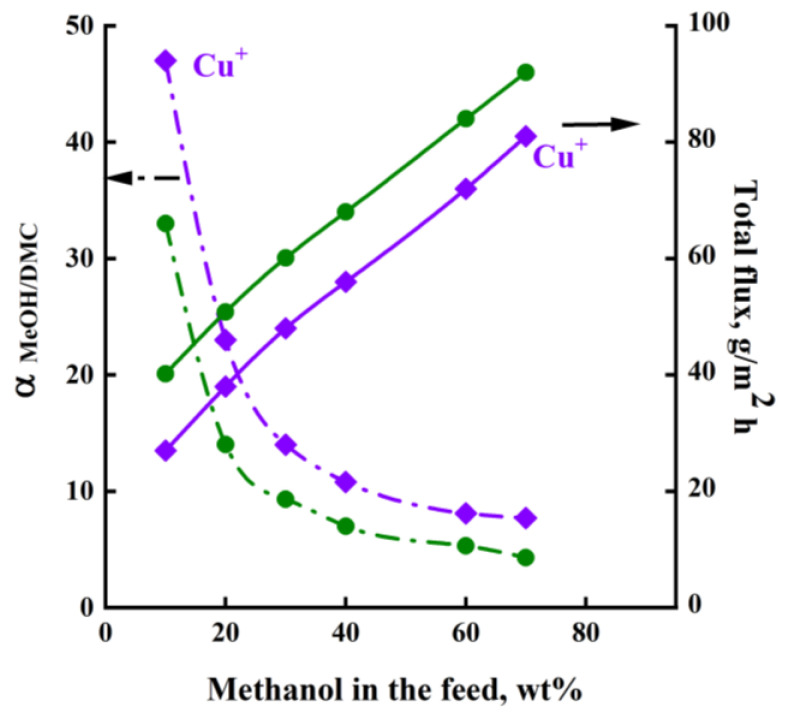
Dependence of separation factor (*α_MeOH/DMC_*) and total flux on methanol concentration in the feed for pervaporation of the MeOH/DMC mixture using the (1) PHI and (2) PHI-Cu(I) membranes, 40 °C.

Figure 9 shows the main transport properties of the PHI and PHI-Cu(I) membranes, namely, the separation factor (*α_MeOH/DMC_*) and the total flux in pervaporation of the MeOH/DMC mixture. An increase in the methanol concentration in the feed leads to an increase in the total flux through both membranes and a decrease in the separation factor. It was found that the total flux through the PHI membrane is higher than that through the PHI-Cu(I) membrane for all the methanol concentrations in the feed. At the same time, the PHI-Cu(I) membrane exhibits a higher separation factor than that of the PHI membrane. 

These results may be due to the fact that the vapor pressure of methanol (414.2 mm Hg) is higher than that of DMC (55.4 mm Hg) [53]; the methanol vaporization is favorable due to its higher driving force. To exclude the effect of the driving force and evaluate intrinsic properties of the penetrant–membrane system, the approach of Baker et al. [47] was used. The normalized transport properties of membranes are permeability and selectivity. Following the procedures described in [47], we calculated the values of the membrane permeability for the individual substances (MeOH and DMC), as well as the selectivity (*β_MeOH/DMC_*) for the PHI and PHI-Cu(I) membranes.

**Figure 10:**
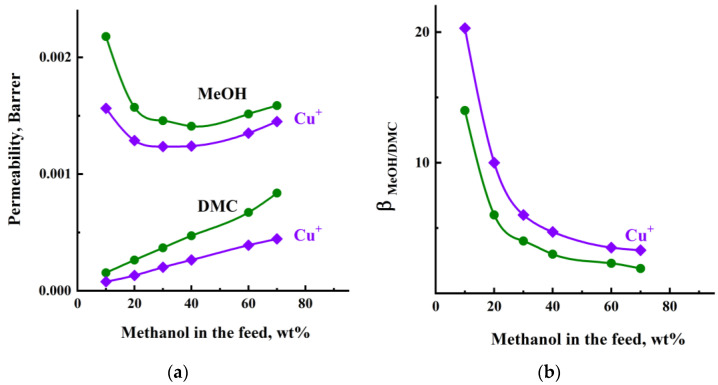
(**a**) Permeability of methanol and DMC and (**b**) selectivity (*β_MeOH/DMC_*) vs. methanol concentration in the feed for pervaporation through the (1, 1′) PHI and (2, 2′) PHI-Cu(I) membranes, 40 °C.

Figure 10a demonstrates the dependences of the MeOH and DMC permeabilities through the PHI and PHI-Cu(I) membranes on the MeOH concentration in the feed. The permeability of both membranes depends on the MeOH concentration in the feed, even when the driving force contribution is excluded. In particular, the permeability of MeOH is high at a low MeOH concentration, but sharply decreases and slowly changes in the range above 20–30 wt% methanol in the feed. The DMC permeability, on the contrary, increases with an increase in the MeOH concentration in the feed, which can be associated with the fact that the membrane becomes more swollen and the penetration of large DMC molecules is facilitated. The permeability of both MeOH and DMC is higher through the PHI membrane as compared to the PHI-Cu(I) membrane.

Figure 10b shows the dependence of the membrane selectivity on the MeOH concentration in the feed. The course of the selectivity curves is similar to that of the separation factor (Figure 8), but the values of selectivity are lower than that of the separation factor. The PHI-Cu(I) membrane exhibits higher values of both the selectivity (*β_MeOH/DMC_*) and separation factor (*α_MeOH/DMC_*) in comparison with the PHI membrane.

**Figure 11:**
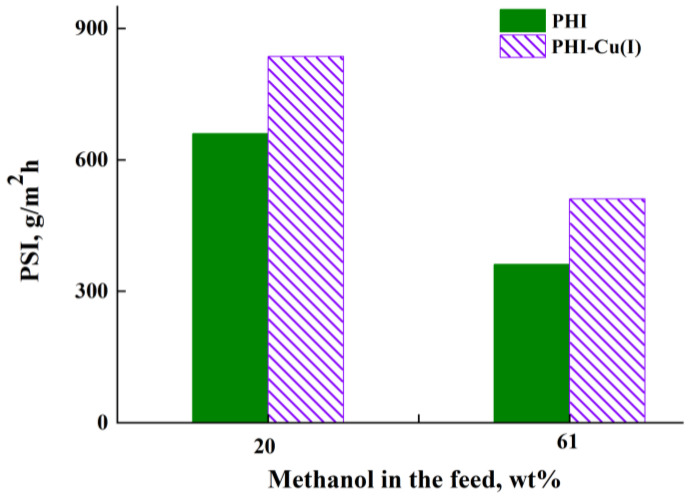
Pervaporation separation index (*PSI*) vs. methanol concentration in the feed for pervaporation through the (1, 1′) PHI and (2, 2′) PHI-Cu(I) membranes, 40 °C.

To appropriately compare the effectiveness of the PHI and PHI-Cu(I) membranes in the separation of the MeOH/DMC mixture, we calculated the *PSI* values, which includes both the parameters (the total flux and separation factor) of the studied membranes. Figure 11 shows the *PSI* values of the membranes in pervaporation of the MeOH/DMC mixture using the feed containing *i*) a small amount of methanol (20 wt%) and *ii*) 61 wt% methanol near azeotropic point. The best transport properties were found for the PHI-Cu(I) membrane in both cases.

## 4. Conclusions

In the present work, poly(2,2′-biquinoline-6,6′-dicarbohydrazide)-co-(bistrimelliteimide) methylene-bisanthranylide (PHI) and its metal–polymer complex PHI-Cu(I) were synthesized using the new monomer 2,2′-biquinoline-6,6′-dicarbohydrazide. Novel PHI and PHI-Cu(I) membranes containing several types of functional groups (hydrazide, carboxyl, amide, and imide fragments) were prepared as dense nonporous films. Comparative study on their structure, physical, mechanical, thermal, and transport properties was carried out. The main mechanical properties, namely the Young’s modulus, the yield stress, and the break stress of both membranes, do not differ significantly, but the ultimate deformation *ε_b_* values differ palpably for PHI (32%) and PHI-Cu(I) (55%) as a result of a non-uniform character of the deformation process for the PHI membrane. The thermal analysis based on the TGA and DTA curves of the PHI and PHI-Cu(I) membranes reveals the peculiarities of the membrane structure and shows the good thermal stability of the membranes in possible conditions of their use (operation) up to 300 °C.

Transport properties of the PHI and PHI-Cu(I) membranes were studied in pervaporation of the MeOH/DMC mixtures in a wide range of feed concentrations, including the composition of the azeotropic mixture: 61 wt% methanol and 39 wt% DMC, at 40 °C. It was found that both membranes produce a permeate enriched with MeOH. It was found that the total flux through the PHI membrane is higher than that through the PHI-Cu(I) membrane for the entire range of methanol concentrations in the feed. At the same time, the PHI-Cu(I) membrane exhibits a higher separation factor than that of the PHI membrane. Evaluation of intrinsic properties of the penetrant–membrane system performed using the Baker approach shows the same trend for permeability and selectivity as for the flux and separation factor for both membranes. Calculation of the pervaporation separation index allows us to conclude that the PHI-Cu(I) membrane exhibits better transport properties as compared with the PHI membrane.

## Figures and Tables

**Table 1:** Mechanical characteristics of the membranes.

Membrane	*E*, (GPa)	*σ_y_*, (MPa)	*σ_b_*, (MPa)	*ε_b_*, (%)
PHI	3.62 ± 0.14	130 ± 4	122 ± 3	32 ± 3
PHI-Cu(I)	3.47 ± 0.19	127 ± 3	117 ± 1	55 ± 2

**Table 2:** Characteristics of thermal stability of the membranes.

Membrane	*τ*_5_, (°C)	*τ*_10_, (°C)	*T*(DTA max), (°C)
PHI	438	484	570
PHI-Cu(I)	372	391	480

**Table 3:** Physical properties of liquids under the study.

Liquid	Mol. Weight, (g/mol)	Density *, (g/cm^3^)	Molar Volume, (cm^3^/mol)	Kinetic Diameter, A^°^	Dipole Moment, (D)	*T_b_*, (°C)
MeOH	32.04	0.7866	40.73	3.8	1.66	64.7
DMC	90.08	1.0635	84.70	6.0	0.93	90.0

* at 298.15 K [14].

**Table 4:** Physical properties of the membranes.

Membrane	Density, (g/cm^3^)	Sorption Degree, (*g_MeOH_*/100 *g_polymer_*)	Diffusion Coefficient of MeOH *D*⋅10^11^, m^2^/min
PHI	1.368	14.1	1.37
PHI-Cu(I)	1.375	12.4	1.18

## Data Availability

The data presented in this study are available on request from the corresponding author.
